# Neuroendokrine Neoplasien

**DOI:** 10.1007/s00292-025-01415-z

**Published:** 2025-02-19

**Authors:** Aziz Chouchane, Konstantin Bräutigam, Aurel Perren

**Affiliations:** 1 Institut für Gewebemedizin und Pathologie, Universität Bern, Bern, Schweiz; 2 Centre for Evolution and Cancer, Institute of Cancer Research, London, Großbritannien

**Keywords:** Klassifikation, Tumorgrading, Immunhistochemie, Lunge, Pankreas, Classification, Tumor grading, Immunohistochemistry, Lung, Pancreas

## Abstract

Neuroendokrine Tumoren (NET) sind eine bunte Gruppe von Neoplasien, die von neuroendokrinen Zellen im gesamten Körper ausgehen. Die NET-Diagnose stellt aufgrund ihrer vielfältigen Erscheinungsformen, Morphologie und biologischen Verhaltensweisen eine besondere Herausforderung dar. Dieser Artikel bietet einen Überblick über die wichtigsten für Allgemeinpathologen relevanten diagnostischen Prinzipien, und betont die Bedeutung eines multidisziplinären Ansatzes, der klinische, radiologische, histopathologische und immunhistochemische Daten integriert. Die Etablierung neuer Marker sowie jüngste Fortschritte in der molekularen Pathologie und die Anwendung von Klassifizierungssystemen werden erörtert, wobei deren Einfluss auf die Prognose und therapeutische Strategien hervorgehoben wird.

## Abstract (en)

Neuroendocrine tumors (NETs) are a diverse group of neoplasms that originate from neuroendocrine cells throughout the body. Diagnosing NETs presents unique challenges due to their varied presentation, morphology, and biological behavior. This article provides an overview of the key diagnostic principles relevant to general pathologists, emphasizing the importance of a multidisciplinary approach that integrates clinical, radiological, histopathological, and immunohistochemical data. The emergence of new markers as well as recent advances in molecular pathology and the application of grading systems are discussed, highlighting their impact on prognosis and therapeutic strategies.

## Lernziele

Nach der Lektüre dieses Beitrags …

- kennen Sie die Morphologie neuroendokriner Tumoren (NET) und wissen um ihr organ-übergreifendes Auftreten.
- kennen Sie die Bedeutung des Proliferationsindex Ki-67 und der Mitosezahl zur Graduierung von neuroendokrinen Neoplasien (NEN).
- sind Ihnen die häufigsten NEN des Magendarmtrakts einschließlich des Pankreas bekannt.
- beherrschen Sie die Einteilung pulmonaler NEN inklusive ihrer Vorläufer.

## Allgemeine Grundsätze

Aufgrund ihrer Eigenschaft, in nahezu allen Organen aufzutreten, sind NET eine häufige Differenzialdiagnose in der täglichen pathologischen Praxis. Das Erkennen ihrer charakteristischen Merkmale und die Anwendung geeigneter diagnostischer Marker sind wichtig, um eine genaue Klassifizierung und Prognostizierung zu gewährleisten. Hierfür ist der Ursprung der Tumoren aus neuroendokrinen Zellen von entscheidender Bedeutung. Diese **epithelialen Ursprungszellen**

epithelialen Ursprungszellen

besitzen sowohl neurale als auch endokrine Eigenschaften und sind in der Lage, Peptidhormone und biogene Amine zu produzieren. Die Sekretion solcher Hormone kann zu einer Vielzahl von klinischen Syndromen führen. Während bei funktionellen NET die **endokrinen Symptome**

endokrinen Symptome

im Vordergrund stehen, ist bei nichtfunktionellen NET der Masseneffekt der Tumoren symptombestimmend. Nichtfunktionelle NET sind zudem immer häufiger Zufallsbefund bei bildgebenden Untersuchungen.

### Merke

Die NET treten organübergreifend auf.

## Histologie

### Typisierung

Zu den typischen morphologischen Merkmalen von NET gehören zytologische und architektonische Kriterien. Zytologisch bestehen NET aus monomorphen Zellen mit rundlichen bis ovalen Kernen mit einem grobkörnigen **„Salz-und-Pfeffer“-Chromatin**

**Abb. 1:**
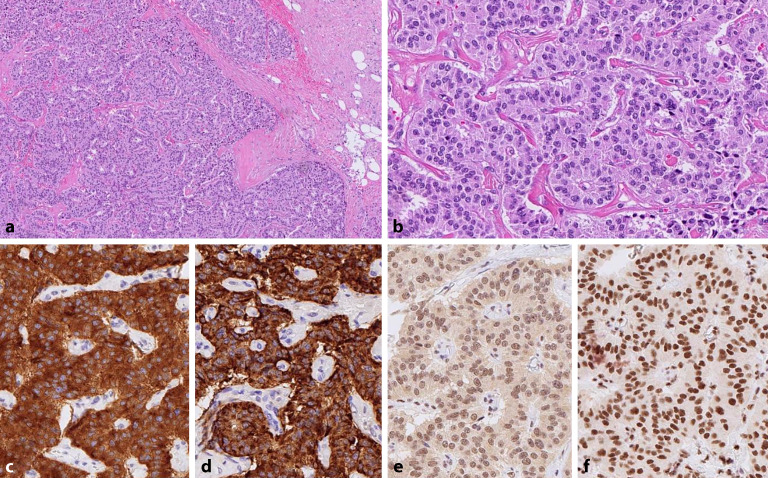
Gut differenzierter neuroendokriner Tumor mit mikrotrabekulärem Wachstumsmuster und hyalinisiertem Stroma (**a**, **b**). Starke homogene Positivität für Chromogranin A (**c**) und Synaptophysin (**d**). Erhaltene nukleäre Expression von DAXX und ATRX (**e**, **f**)

„Salz-und-Pfeffer“-Chromatin

und manchmal exzentrischem teils plasmazytoidem Zytoplasma (Abb. 1a, b). Gelegentlich können zelluläre oder nukleare Atypien mit erhöhter Kernpleomorphie beobachtet werden, die mit chromosomaler Instabilität einhergehen und als **„endokrine Atypie“**

„endokrine Atypie“

bezeichnet werden. Klassischerweise werden 4 wesentliche architektonische Wachstumsmuster von NET beschrieben: nestförmig, trabekulär, pseudoglandulär und diffus. Die NET aus verschiedenen Ursprungszellen und verschiedenen Organen können mit einer Tendenz zu einem bestimmten **Wachstumstyp**

Wachstumstyp

assoziiert sein [1, 2], was manchmal bei der Differenzialdiagnose von NET mit unbekanntem Ursprung hilfreich sein kann. Alle diese morphologischen Muster werden als „gut differenziert“ („well differentiated“) zusammengefasst.

Im Gegensatz hierzu stehen die „gering differenzierten“ („poorly differentiated“) **neuroendokrinen Karzinome**

neuroendokrine Karzinome

(NEC). Diese hoch aggressiven Tumoren stammen wahrscheinlich nicht von neuroendokrinen Zellen ab, sondern weisen eine aberrante neuroendokrine Differenzierung auf und sind mit den Adenokarzinomen verwandt. Gering differenzierte NEC haben eine andere Morphologie als NET und häufig reichen histologische Merkmale aus, um beide zu unterscheiden. Die NEC werden meistens in **2 Gruppen**

2 Gruppen

unterteilt: großzellige NEC (LCNEC) oder kleinzellige NEC (SCNEC). Außerhalb der Lunge ist der großzellige Phänotyp etwas häufiger anzutreffen, aber in etwa 30 % der nichtpulmonalen NEC ist eine klare Zuordnung zu diesen 2 Typen nicht gegeben. In der Regel treten NEC als solide Massen mit polygonalen Zellen, großen Kernen und hoher mitotischer Aktivität auf. Die Nester sind größer und es finden sich solide Areale. Es werden häufiger „landkartenartige“ Nekrosen beobachtet. Der kleinzellige Phänotyp zeichnet sich durch Zellen mit einer großen Kern-Zytoplasma-Ratio, einer Kernverformung und nicht selten durch einen **„Crush“-Artefakt**

„Crush“-Artefakt

(„Azzopardi-Phänomen“) aus.

### Staging

Das Tumorstadium spielt bei der Bestimmung der Prognose von NET eine wichtige Rolle. Während für NEC das Staging-System der Union Internationale Contre le Cancer (UICC) für Adenokarzinome angewandt wird, bestehen für NET organspezifische **TNM-Systeme**

TNM-Systeme.

Es hat sich gezeigt, dass invasiv-wachsende Tumoren einen deutlich ungünstigeren und schnelleren Krankheitsverlauf haben als gut umschriebene oder abgekapselte Tumoren [3, 4].

## Immunhistochemie

### Neuroendokrine Differenzierung

Die Immunhistochemie (IHC) spielt bei der Bestätigung der neuroendokrinen Differenzierung und der **Subklassifizierung**

Subklassifizierung

von NET eine entscheidende Rolle. Die am häufigsten verwendete Marker sind **Chromogranin A**

Chromogranin A

und Synaptophysin, die beide spezifisch für neuroendokrine Zellen sind (Abb. 1c, d). Während Synaptophysin sehr sensitiv ist, ist Chromogranin A spezifischer. In den letzten Jahren hat sich das insulinomassoziierte Protein 1 (INSM1) als neuer und hochempfindlicher Marker für die **neuroendokrine Differenzierung**

neuroendokrine Differenzierung

herausgestellt. Hierbei handelt es sich um einen Transkriptionsfaktor mit für die Entwicklung neuroendokriner Zellen entscheidender Bedeutung, der in NET und NEC eine robuste Expression mit kombinierter hoher Sensitivität und Spezifizität aufweist [5]. Aufgrund mangelhafter Spezifität sollte CD56 nicht eingesetzt werden [6]. In 70 % der ösophagealen NEC wird TTF‑1 exprimiert, was jedoch zur Unterscheidung von anderen viszeralen NEC nicht hilfreich ist, da die meisten NEC (mit Ausnahme von Merkel-Zell-Karzinomen) TTF‑1 exprimieren.

#### Merke

Einschlägige immunhistochemische Untersuchungen helfen in der Bestätigung der Einschätzung einer Neoplasie als neuroendokrinen Ursprungs.

### *TP53* und *RB1*

Die Tumorsuppressorgene *TP53* und *RB1* spielen in der Diagnostik von hochgradigen NEN eine wichtige Rolle, insbesondere in den Fällen, in denen die Morphologie zur Unterscheidung zwischen einem NET G3 und einem NEC nicht ausreichend ist. Sie sind in bis zu 70 % der NEC mutiert/inaktiviert, während sie in den meisten NET als Wildtyp erhalten sind [7]. Ein kompletter Verlust der nukleären Expression von Rb1 reflektiert die **Geninaktivierung**

Geninaktivierung,

wobei stromale Zellen oder benachbartes Endothel als positive Kontrolle dienen (Abb. 3). Da diese sowohl durch inaktivierende Mutationen als auch durch **epigenetische Inaktivierung**

epigenetische Inaktivierung

stattfindet, ist die Immunhistochemie sensitiver als klassische Next-Generation-Sequencing(NGS)-Untersuchungen. Die *RB1*-inaktivierten Tumoren sprechen besser auf **platinbasierte Chemotherapien**

platinbasierte Chemotherapien

an [8]. Wie bei anderen Organen oder Tumoren kann die Interpretation der immunhistochemischen Färbung von p53 in einigen Fällen problematisch sein, aber im Prinzip kann der vollständige Verlust der nukleären Expression oder eine auffällige Überexpression als ein pathologischer Befund mit einer hohen Wahrscheinlichkeit für das Vorliegen einer inaktivierenden Mutation interpretiert werden. Die definitive Einordnung von NET G3 und NEC muss allerdings in erster Linie aufgrund histologischer **Wachstumsmuster**

Wachstumsmuster

erfolgen, da auch wenige NET G3 eine pathologische Expression von p53 (10 %) und Rb1 (< 5 %) zeigen können [9].

### Somatostatinrezeptor 2A 

Die Somatostatinrezeptoren (SSTR), insbesondere SSTR2A, sind im Kontext von NET weniger von diagnostischer als von prognostischer und therapeutischer Bedeutung. **Somatostatinanaloga**

Somatostatinanaloga

können zur Suppression von endokrinen Symptomen und zur Verlangsamung des Tumorwachstums eingesetzt werden. Die Kombination von Analoga (oder Antagonisten) mit radioaktiven Isotopen wird zur **Peptid-Rezeptor-Radiotherapie**

Peptid-Rezeptor-Radiotherapie

(PRRT) eingesetzt. Die Therapieindikation ist an einen nuklearmedizinischen Nachweis des Somatostatinrezeptors gekoppelt, diese korreliert jedoch gut mit der immunhistochemischen Expression. Bei positiver Expression wird ein **Intensitätsscore**

**Abb. 2:**
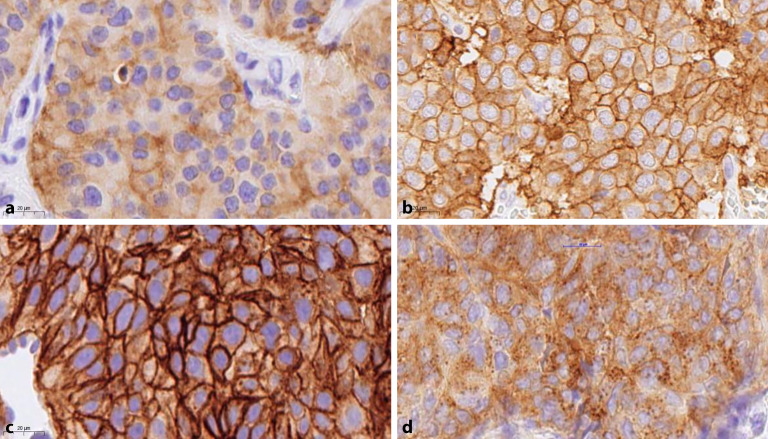
Immunhistochemische Färbung von Somatostatinrezeptor 2A (SSTR2A). **a** Intensität: +1. **b** Intensität: +2. **c** Intensität: +3. **d** Beispiel für eine „internalisierte“, nichtmembranöse Färbung. (Sollte nicht als positiv interpretiert werden.)

Intensitätsscore

von 1–3 angegeben (Abb. 2). Bei einer membranösen Positivität von 10 % der Tumorzellen werden NET auch in den entsprechenden **Positronenemissionstomographien**

Positronenemissionstomographien

(PET) detektiert [10].

#### Cave

Wichtig zu wissen ist, dass eine negative SSTR2A-Immunhistochemie in der Biopsie nicht automatisch die Notwendigkeit einer SSTR-basierten Bildgebung ausschließt, da die SSTR2-Expression in Tumoren häufig heterogen ist und je nach eingesetzter Substanz auch SSTR5 Bindung vorkommt.

### *DAXX/ATRX* und *Menin*

Die Gene *DAXX* und *ATRX* kodieren für Proteine, die an der Aufrechterhaltung der **chromosomalen Stabilität**

chromosomalen Stabilität

beteiligt sind, insbesondere in der Telomer- und Zentromerregion der Chromosomen. Ihre Rolle bei der Tumorprogression ist im Zusammenhang mit den NET des Pankreas wichtig, bei denen somatische Mutationen in diesen Genen mit einem aggressiveren Krankheitsverlauf und chromosomaler Instabilität korreliert sind [11]. Der Verlust der nukleären immunhistochemischen Expression (Abb. 1e, f) eines dieser Proteine ist ein Hinweis auf eine pathogene Mutation und ein **prognostischer Marker**

prognostischer Marker,

der in Zukunft in die Standarduntersuchung von pankreatischen NET aufgenommen werden könnte. *Menin* ist bei pankreatischen, duodenalen, gastrischen und pulmonalen NET mutiert, und zwar sowohl im familiären als auch im sporadischen Setting. *Menin*-Mutationen haben keine prognostische Bedeutung. Im Gegensatz zu den NET weisen NEC keine Mutationen in *Menin, DAXX* oder *ATRX* auf.

### Graduierung und Proliferationsindex

Die Einstufung von NET basierend auf der Mitosezahl und dem **Ki-67-Proliferationsindex**

Ki-67-Proliferationsindex

ist für die Beurteilung der Tumoraggressivität und des Therapiealgorithmus entscheidend. Die aktuellen Richtlinien der Weltgesundheitsorganisation (WHO) besagen, dass die Ki-67-Bewertung durch **manuelles Zählen**

manuelles Zählen

auf einem Ausdruck, der mindestens 500 neoplastische Zellen aus den Regionen mit der höchsten Markierung (Hotspots) umfasst, genauer ist als die visuelle Schätzung [12]. Im Rahmen der Digitalisierung werden vermehrt **computerunterstützte Zählverfahren**

computerunterstützte Zählverfahren

eingesetzt. Für die Graduierung von NET wurden **Ki-67-Schwellenwerte**

Ki-67-Schwellenwerte

von 3 und 20 % definiert, das Rückfallrisiko nimmt jedoch kontinuierlich mit zunehmendem Ki-67 Index zu [13]. Daher ist für den Onkologen die Angabe der prozentualen Proliferationsrate ein wichtiges **Entscheidungskriterium**

**Abb. 3:**
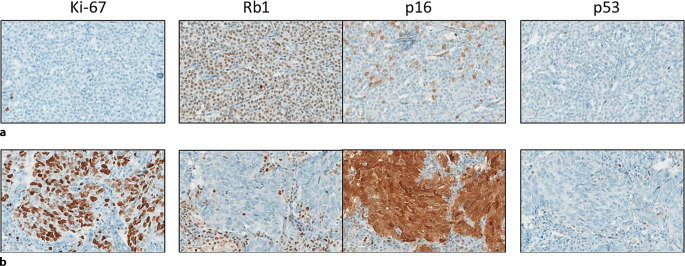
Immunhistochemische Färbung von Ki-67, Rb1, p16 und p53 . **a** Gutdifferenzierter neuroendokriner Tumor (NET) mit erhaltener Rb1-Expression, Wildtyp-p53-Muster, keiner p16 ‚Block-like‘ Positivität und niedrigem Ki-67 (< 1 %). **b** Neuroendokrines Karzinom (NEC) mit hoher Expression von Ki-67 (> 60 %) und Rb1, p53-Expressionsverlust sowie starker p16-Positivität

Entscheidungskriterium

bei der Therapiewahl. Tumoren mit einem Ki-67-Index von ≤ 3 % werden als G1 klassifiziert, während diejenigen mit einem Ki-67-Index zwischen 3 und 20 % als G2 eingestuft werden. Tumoren mit einem Ki-67-Index ≥ 20 % werden als NET G3 klassifiziert (Abb. 3). In seltenen Fällen, in denen NET mit sehr hoher Proliferationsaktivität schwer von NEC zu unterscheiden sind, ist ein **integrativer Ansatz**

integrativer Ansatz

unter Einbeziehung von Histomorphologie, Immunhistochemie und, in einigen Fällen, molekularer Analyse entscheidend für eine genaue Diagnose. Diese Unterscheidung ist von großer Bedeutung, da sich die Behandlungsregime für metastasierte NET G3 und NEC erheblich unterscheiden [14].

#### Merke

Der Ki-67-Proliferationsindex ist für die Graduierung und prognostische Stratifizierung vieler NEN essenziell.

## Organspezifische Manifestationen von neuroendokrinen Tumoren

### Gastroenteropankreatische neuroendokrine Tumoren

Der Gastrointestinaltrakt ist der häufigste Entstehungsort von NET, die ein breites Spektrum von Entitäten namens gastroenteropankreatische NET (GEP-NET) umfassen. Diese Tumoren können im gesamten Gastrointestinaltrakt entstehen, insbesondere im Bereich des Magens, des Dünndarms, der Bauchspeicheldrüse und des Rektums, und weisen jeweils spezifische klinische und pathologische Merkmale auf.

#### Ösophagus

Im Ösophagus sind hochdifferenzierte NET eine Rarität (< 10 %), häufiger sind NEC oder gemischte neuroendokrine-nichtneuroendokrine Neoplasien (MiNEN) mit Kombinationen von NEC mit Adeno- oder Plattenepithelkarzinomen. Die wichtigste Differenzialdiagnose für ein ösophageales NEC ist ein Plattenepithelkarzinom, insbesondere vom **basaloiden Typ**

basaloiden Typ.

Synaptophysin und INSM1 sind in den meisten Fällen exprimiert, während Chromogranin A eine weniger konsistente Expression aufweist (60 %).

#### Magen

Gastrale NET können von mehreren Ursprungszellen abstammen. Tumoren, die aus Enterochromaffin-like(ECL)-Zellen entstehen, werden aufgrund ihrer Ätiologie und histologischen Merkmale in die Typen 1, 2, 3 und (provisorisch) 4 und 5 unterteilt. Wichtig für die Prognose ist der Zusammenhang mit **Hypergastrinämie**

Hypergastrinämie,

der bei Typ 1 und 2 zentral ist. Die ECL-Zell-NET vom Typ 1 entstehen auf dem Boden einer chronisch-atrophen **Gastritis**

Gastritis

mit autoimmuner oder *Helicobacter-pylori*-assoziierter Genese, die zur Hypergastrinämie und **ECL-Zell-Hyperplasie**

ECL-Zell-Hyperplasie

führt. Sie sind der häufigste Typ und machen etwa 80–90 % der ECL-Zell-NET aus. Typ 2 ist durch die Hypergastrinämie des **Zollinger-Ellison Syndroms**

Zollinger-Ellison Syndroms

bei mit multipler endokriner Neoplasie (MEN) 1 assoziierten (duodenalen) Gastrinomen bedingt und weist etwas häufiger **Lymphknotenmetastasen**

Lymphknotenmetastasen

auf. Aufgrund der weit verbreiteten durch Protonenpumpeninhibitoren (PPI) induzierten Hypergastrinämie wurde in letzter Zeit ein neuer, provisorischer Typ (ECL-Zell-NET vom Typ 5) beschrieben, der ebenfalls multifokal ist und mit einer PPI-assoziierten Hypertrophie der Parietalzellen einhergeht. Eine ähnliche Entität, die mit einem intrinsischen Defekt der Säuresekretion aus den Parietalzellen zusammenhängt, ist mit Hypergastrinämie, Achlorhydrie und Parietalzellhyperplasie verbunden [15]. Somit ist bei allen gastrininduzierten Magen-NET die Untersuchung von normaler **Korpusmukosa**

Korpusmukosa

entscheidend. Während bei Typ 1 eine Atrophie vorliegt, sind bei Typ 3 und 5 die Hyperplasie/Hypertrophie der Belegzellen neben der ECL-Zell-Hyperplasie diagnostisch wichtig. Der Typ 3 ist in der Regel solitär (unifokal), tritt in normaler Magenschleimhaut auf und ist klinisch aggressiver, sobald die Magenmuskulatur infiltriert wird. Typ 4 Magen NETs sind hereditär, und sind mit einer inaktivierenden APT4A Mutation verbunden. Zu den anderen NEN des Magens gehören EC-Zell- und Gastrin(G)-Zell-Tumoren sowie die NEC, die etwa 20 % aller NEN des Magens ausmachen. Wie im übrigen Körper werden sie in großzellige oder kleinzellige NEC unterteilt, ohne dass eine spezifische höhere Prävalenz eines der beiden Subtypen besteht.

#### Duodenum

Duodenale NET produzieren häufig Gastrin oder Somatostatin [16]. Etwa die Hälfte der gastrinproduzierenden duodenalen NET geht mit einem Zollinger-Ellison-Syndrom mit multiplen Ulzera und **Durchfällen**

Durchfällen

einher. Somatostatinproduzierende NET sind meist ohne hormonelle Symptome, können aber aufgrund ihrer periampullären Lage mit **Ikterus**

Ikterus

einhergehen. Sie sind histologisch durch ein pseudoglanduläres Muster mit gelegentlichen Psammomkörpern charakterisiert. Kombinierte Gangliozytome/NET treten nur im Duodenum auf („CoGNET“). Im Bereich der Ampulla sind auch NEC beschrieben.

#### Dünndarm

Dünndarm-NET (SI-NET) treten meist im Ileum, seltener im Jejunum auf. Jejunoileale NET sind häufig multifokal (ein Drittel der Fälle), was jedoch nicht unbedingt mit einem aggressiveren Verlauf korreliert [17]. Sie zeigen oft eine Architektur in Nestern und produzieren **Serotonin**

Serotonin.

Die meisten SI-NET exprimieren SSTR2A und den Transkriptionsfaktor CDX2, im Gegensatz zu pankreatischen NET, die Islet‑1 exprimieren, oder NET der Lunge bzw. Schilddrüse mit TTF-1-Expression. Jejunoileale NEC sind eine Rarität.

#### Kolon, Rektum und Anus

Im Kolon können überall NEN auftreten, wobei (mit Ausnahme des Appendix) das Rektum die häufigste Lokalisation darstellt. Die EC-Zell-Tumoren zeigen die typische genestete Morphologie, während L‑Zell-Tumoren (häufig im Rektum) typischerweise eine trabekuläre oder **pseudoglanduläre Morphologie**

pseudoglanduläre Morphologie

aufweisen. Die NET des Kolons sind variabel SSTR2A-positiv. Der für die kolorektale Schleimhaut spezifische Transkriptionsregulator SATB2 wird nachweislich auch in den meisten NET des Rektums und des Appendix exprimiert [18]. Rektale L‑Zell-NET sind auch häufig negativ für Chromogranin A und CDX2 (Tab. 1). Die NEC in dieser Region zeigen die typischen Merkmale entweder kleinzelliger oder großzelliger NEC.

**Tab. 1:** Immunhistochemische Expression von Transkriptionsfaktoren in gut differenzierten (G1 bis G3) neuroendokrinen Tumoren

Marker	Dünndarm	Rektum/Appendix	Magen	Lunge	Pankreas
Chromogranin A, Synaptophysin, INSM1	+	±	+	+	+
CDX2	+	±	+	–	–
TTF‑1	–	–	–	+	–
Islet1	–	–	–	±	+
SATB2	–	+	–	–	–
OTP	–	–	–	+	–
Serotonin	+	–	–	–	–

#### Appendix vermiformis

Appendix-NET treten in den meisten Fällen in der Spitze der Appendix auf und werden meist zufällig nach einer Operation wegen akuter Appendizitis entdeckt. Makroskopisch erscheinen sie als gut abgegrenzte **gelbe Läsionen**

gelbe Läsionen

mit meist einem Durchmesser von < 1 cm. Morphologisch kann der Tumor den SI-NET ähneln (entsprechend EC-Zell-NET) oder trabekulär/tubulär aufgebaut sein (L-Zell-NET). Obwohl diese beiden NET mit unterschiedlicher Häufigkeit Lymphknotenmetastasen aufweisen, scheinen diese klinisch nicht relevant zu sein: Eine onkologische Hemikolektomie bei Appendix-NET mit einem Durchmesser von 1–2 cm liefert keine überlegenen Behandlungsergebnisse, was den Weg für weniger aggressive Ansätze und eine bessere Patientensicherheit ebnet [19].

### Pankreatische neuroendokrine Neoplasien

Pankreatische NET (PanNET) machen etwa 2–5 % der Pankreastumoren und 11 % aller NET im Körper aus. Traditionell werden sie basierend auf ihrer Fähigkeit, Hormone zu sezernieren, in 2 Gruppen unterteilt: funktionelle (hormonproduzierende) PanNET und nichtfunktionelle (NF-)PanNET. Ein positiver immunhistochemischer Nachweis von Hormonen, wie pankreatisches Polypeptid (PP) und **Glukagon**

Glukagon,

bedeutet nicht, dass der Patient klinische Symptome aufweist. Auch pankreatische NEC (PanNEC) können auftreten.

#### Nichtfunktionelle PanNET

Die NF-PanNET sind per Definition größer als 0,5 cm. Tumoren < 0,5 cm werden als Mikrotumoren (früher „Mikroadenome“) bezeichnet. Die NF-PanNET sind in der Regel langsam wachsende Tumoren. Aufgrund ihrer Tendenz, lange unbemerkt zu bleiben, befinden sich jedoch etwa 30 % der Patienten bei Diagnose bereits im metastasierten Stadium. Morphologisch zeigen sie das typische Spektrum endokriner Wachstumsmuster, am häufigsten trabekulär, nestförmig oder solide, mit unterschiedlich stark ausgeprägter **Sklerose**

Sklerose

und Fibrose. Diese Tumoren sind positiv für neuroendokrine Marker (Chromogranin A, Synaptophysin, INSM1). Im Fall einer metastasierten Erkrankung mit unbekanntem Primärtumor kann Islet1 verwendet werden, fehlende Expression schließt eine pankreatische Herkunft allerdings nicht automatisch aus [20]. Weniger häufig, aber wichtig zu kennen, sind die onkozytären und **klarzelligen Subtypen**

klarzelligen Subtypen.

Die Differenzialdiagnose von PanNET umfasst:

- die solide papilläre Neoplasie (SPN; nukleäres β‑Catenin),
- Azinuszellkarzinome, die positiv für Trypsin und Bcl-10 sind (ausschließlich Bcl-10-Klon 331.1, der eine Kreuzreaktivität mit dem exokrinen Pankreasenzym Carboxylesterhydrolase aufweist [21]) und seltener
- Paragangliome des Pankreas (negativ für Zytokeratine, positiv für Pax6 und GATA 3).

Bei hellzelligem PanNET muss an die Möglichkeit einer Metastase eines Nierenzellkarzinoms gedacht werden (Tab. 2).

**Tab. 2:** Differenzialdiagnose und immunhistochemischer Algorithmus von pankreatischen neuroendokrinen Tumoren (PanNET)

Differenzialdiagnose	Synaptophysin	Chromogranin A	INSM1	Zytokeratin	Bcl10	β‑Catenin
PanNET	+	+	+	+	–	− selten + (nukleär)
PanNEC	+	+	+	+	–	–
Paragangliom	+	+	+	–	–	–
Metastase RCC	− selten +	–	–	+	–	–
SPN	±	–	–	+	–	+ (nukleär)
Azinuszellkarzinom	±	–	–	+	+	–

*NEC* neuroendokrines Karzinom, *NET* neuroendokrine Tumor, *RCC* Nierenzellkarzinom, *SPN *solide papilläre Neoplasie

#### Funktionelle PanNET

Funktionelle PanNET sind eine heterogene Gruppe von Tumoren. Insulinome machen etwa 80 % der funktionierenden PanNET aus und können sowohl sporadisch als auch – seltener – familiär bedingt auftreten. Glucagonome und **VIPome**

VIPome

(VIP: vasoaktives intestinales Peptid) sind selten. Histologisch können sie solide, trabekulär und in einigen Fällen zystisch (wie Glucagonome) imponieren. Die große Mehrheit sind niedriggradige Tumoren und metastasieren in 90 % der Fälle nicht. Bei den meisten anderen funktionellen PanNET treten häufig Metastasen auf. Die Existenz von klinisch funktionellen Somatostatinomen ist umstritten [22]. Die PanNET können (insbesondere unter Therapie) eine Progression im Hinblick auf die Proliferationsrate (Ki-67) und folglich den Tumorgrad durchlaufen. Fast 50 % der NET G3 zeigen ihren Ursprung im Pankreas [23].

#### PanNEC

Die PanNEC sind seltener als PanNET. Sie sind mit keinem endokrinen Tumorsyndrom verbunden. Histologisch weisen sie die typischen malignen Zeichen von groß- oder kleinzelligen NEC auf, wobei sie häufig einen sehr hohen Ki-67-Index (mindestens 60–80 %) und eine hohe **mitotische Aktivität**

mitotische Aktivität

aufweisen. Wenn eine zweite nichtneuroendokrine Komponente (z. B. Adenokarzinomanteile) im Tumor vorhanden ist, muss diese mindestens 30 % des Tumors ausmachen, damit dieser als MiNEN klassifiziert wird. In mehreren genetischen und epigenetischen Studien wurde nachgewiesen, dass PanNEC im Vergleich mit gut differenzierten NET ein ähnliches Mutations- und Transkriptomprofil aufweisen wie pankreatische duktale Adenokarzinome (PDAC) und Azinuszellkarzinome. In seltenen Fällen haben sich bei einigen NET G3 auch *RB1*- und *TP53*-Mutationen sowie hochgradige histomorphologische Merkmale gezeigt. Solche Tumoren werden als NET G3 mit einer NEC-ähnlichen **Transformation**

Transformation

bezeichnet und sprechen bisher nicht wie die klassischen NEC auf platinbasierte Therapien an [24, 25].

### Pulmonale neuroendokrine Neoplasien

Die pulmonalen NEN werden ebenfalls in pulmonale NET und pulmonale NEC unterteilt.

#### Pulmonale NET

Zur Gruppe der pulmonalen NET gehören das typische **Karzinoid**

Karzinoid

(G1) und das atypische Karzinoid (G2). Ein kleiner Teil der Lungen-NET weist zahlreiche Vorläuferläsionen (präinvasiv) auf, dies kann im Rahmen einer MEN1 oder häufiger einer diffusen idiopathischen pulmonalen neuroendokrine Zellhyperplasie (DIPNECH) beobachtet werden. Präneoplastische DIPNECH-Läsionen sind sehr indolent und nur wenige entwickeln sich weiter zu NET [26]. Deshalb werden diese klinisch beobachtet. Bei pulmonalen NET bestimmt derzeit noch die Mitosenzahl den Grad, auch wenn der Ki-67-Index zusätzlich angegeben werden sollte. Eine Anzahl von < 2 Mitosen/2 mm^2^ und das Fehlen von Nekrosen definieren ein typisches Karzinoid. Eine Anzahl von > 2 Mitosen/2 mm^2^ und/oder das Vorhandensein von Nekrosen definieren ein atypisches Karzinoid. Diese Tumoren sind für die neuroendokrinen Marker und teils für TTF‑1 positiv. **Orthopedia-Homeobox-Protein**

Orthopedia-Homeobox-Protein

(OTP) hat sich auch als sensitiver (≈ 80 %) und sehr spezifischer Marker (> 99 %) für primäre Lungen-NET erwiesen [27]. Bei der Gradierung eines Karzinoidtumors anhand einer Biopsie ist der Ki-67-Index wichtig, es besteht jedoch wie immer bei Biopsien das Risiko einer Fehleinschätzung der Gradierung.

#### Pulmonale NEC

Die pulmonalen NEC werden in den großzelligen- (LC) und den kleinzelligen (SC) Subtyp unterteilt. Aufgrund der Assoziation zum **Rauchen**

Rauchen

sind kleinzellige (neuroendokrine) Lungenkarzinome (SCLC) mit etwa 15 % der Lungentumoren viel häufiger als im GEP-System. Das SCLC neigt dazu, sich zentral in den großen Atemwegen zu manifestieren, im Gegensatz zu den LCNEC, die eher in der Peripherie vorkommen. Bei einigen nichtkleinzelligen Lungenkarzinomen (NSCLC; insbesondere EGFR-mutiertes Adenokarzinom) kann eine **Transdifferenzierung**

Transdifferenzierung

in einen SCLC als Resistenzmechanismus auftreten. Die SCLC zeigen wenig Zytoplasma, schlecht abgegrenzte Zellgrenzen und ein „Salz-und-Pfeffer“-Chromatin. Da die Zellen dicht gepackt sind, können sie bei der Biopsie deutliche **Quetschartefakte**

Quetschartefakte

aufweisen. Dies kann die Diagnose erschweren, hier ist der hohe Proliferationsindex manchmal die einzige Abgrenzung zum NET. Molekular sind **transkriptionelle Subtypen**

transkriptionelle Subtypen

(*ASCL1, NEUROD1, POU2F3* und *YAP1*) charakterisiert worden, wobei diese möglicherweise mit Therapieansprechen korrelieren [28].

## Fazit für die Praxis

- Neuroendokrine Tumoren (NET) können in verschiedenen Organen des Körpers als Metastasen oder Primärtumoren auftreten und sollten bei der Differenzialdiagnose berücksichtigt werden.
- Neuroendokrine Marker, Ki-67 und Somatostatinrezeptoren (SSTR) 2 sollten bei jeder Beurteilung eines NET untersucht werden.
- Bei einem NET mit unbekanntem Primärtumor können u. a. die verschiedenen Transkriptionsfaktoren untersucht werden, um die Lokalisierung des Primärtumors näher zu bestimmen.

## CME-Fragebogen

Welche der folgenden Eigenschaften ist *am wenigsten* typisch für eine neuroendokrine Morphologie?

Prominente Nukleolen

Exzentrisches Zytoplasma

Rundliche bis ovale Zellkerne

„Salz-und-Pfeffer“-Chromatin

Spindelige Zellform

Welcher Plattenepithelkarzinomsubtyp ist ein starker Imitator des neuroendokrinen Karzinoms des Ösophagus?

Basaloider Subtyp

Plattenepithelkarzinom, NOS

Spindelzelliger Subtyp

Verruköser Subtyp

Undifferenzierter Subtyp

Welche Aussage trifft zu?

Azinuszellkarzinome zeigen eine nukleäre β‑Catenin-Expression.

Insulinome treten zumeist sporadisch auf.

Nierenzellkarzinome sind eine Differenzialdiagnose des hellzelligen pankreatischen neuroendokrinen Tumors (PanNET)

Pankreatische neuroendokrine Karzinome (NEC) zeigen in der Regel ein Ki-67 von 20–30 %

VIPome des Pankreas sind häufiger als Insulinome

Welches Kriterium differenziert das atypische vom typischen Karzinoid der Lunge?

Expression von CD56

Ki-67 > 10 %

Lymphgefäßinvasion

Nekrose

Trabekuläres Wachstum

Welche Aussage trifft auf kleinzellige Lungenkarzinome (SCLC) zu?

Der Proliferationsindex ist oft relativ niedrig.

Oft sind betroffene Patienten Raucher.

Quetschartefakte sind in SCLC selten.

Die SCLC sind häufiger als Plattenepithelkarzinome der Lunge.

Die SCLC zeigen reichlich Zytoplasma.

Was gilt für pankreatische neuroendokrine Karzinome (PanNEC)?

Der Proliferationsindex liegt oft unter 15 %.

Die mitotische Aktivität ist gewöhnlich hoch.

Lebensgewohnheiten haben einen signifikanten Einfluss auf die Entstehung.

Sie treten vorwiegend syndromal auf.

Sie sind häufiger als pankreatische neuroendokrine Tumoren (PanNET).

Ein 54-jähriger Mann stellt sich mit unklaren Oberbauchbeschwerden und Gewichtsverlust in der Klinik vor. Bildgebend zeigt sich ein gut abgrenzbarer, hypervaskularisierter Tumor im Pankreaskopf. Eine Feinnadelbiopsie wird durchgeführt und histologisch zeigt sich eine solide, azinäre Zellproliferation mit granulärem eosinophilem Zytoplasma. Sie vermuten ein Azinuszellkarzinom. Welchen immunhistochemischen Marker wählen Sie, um Ihren Verdacht zu untermauern?

β-Catenin

GATA3

Ki-67

Panzytokeratin

Trypsin

Was gilt für die neuroendokrinen Tumoren (NET) der Appendix vermiformis (aNET)?

Für gewöhnlich beträgt der Durchmesser mehr als 2 cm.

Makroskopisch ist ein oftmals gelbes Kolorit typisch.

Sie sind negativ für Synaptophysin.

Sie sind meist in der Appendixbasis, zökumnah, lokalisiert.

Trabekuläre Wachstumsmuster sind untypisch.

Ein 62-jähriger Mann stellt sich mit rezidivierender Diarrhö, abdominellen Schmerzen und Gewichtsverlust in der Klinik vor. Bildgebend zeigt sich ein hypervaskularisierter Tumor im Dünndarm mit multiplen Lebermetastasen. Eine Biopsie des Leberherds zeigt histologisch einen gut differenzierten neuroendokrinen Tumor (neuroendokriner Tumor [NET] G2) mit typischer „Salz-und-Pfeffer“-Chromatinstruktur. Zur weiteren Diagnostik und Therapiekontrolle soll eine Somatostatinrezeptors(SSTR)-Szintigraphie erfolgen. Welche Aussage bezüglich SSTR, insbesondere SSTR2 trifft am ehesten zu?

Der immunhistochemische und nuklearmedizinische Nachweis korrelieren miteinander.

Sie sind pathognomonisch für neuroendokrine Tumoren.

Eine hohe SSTR2-Expression korreliert immer mit einer besseren Prognose.

Die SSTR2-Expression ist unabhängig von der Tumordifferenzierung.

Somatostatinanaloga sind zur Suppression von endokrinen Symptomen wenig wirksam.

Ein 58-jähriger Mann stellt sich mit unklaren abdominellen Schmerzen und Gewichtsverlust in der Klinik vor. Eine Bildgebung zeigt einen tumorösen Befund im Pankreas. Eine Biopsie ergibt histologisch eine neuroendokrine Neoplasie mit typischer Zellmorphologie und „Salz-und-Pfeffer“-Chromatin. Der Proliferationsindex Ki-67 beträgt 25 %. Welche Aussage zum Ki-67-Index bei neuroendokrinen Neoplasien trifft am ehesten zu?

Für die Therapiestratifizierung ist der Proliferationsindex Ki-67 unerheblich.

Für die Prognosestratifizierung ist der Proliferationsindex Ki-67 wenig aussagekräftig.

Der Ki-67-Index wird in den am stärksten proliferierenden Bereichen des Tumors (Hotspotregion) bestimmt.

Neuroendokrine Karzinome weisen typischerweise ein relativ geringes prozentuales Ki-67 auf.

Visuelles Schätzen von Ki-67 ist ausreichend objektiv und reproduzierbar.
